# Clinical relevance of gallbladder wall thickening for dengue severity: A cross-sectional study

**DOI:** 10.1371/journal.pone.0218939

**Published:** 2019-08-30

**Authors:** Michel de Araújo Tavares, Guilherme Augusto Pivoto João, Michele Souza Bastos, João Bosco Lima Gimaque, Anne Cristina Gomes Almeida, Thanh Thu Ngo, Cecilia Bahamon, Djane Clarys Baia-da-Silva, Wuelton Marcelo Monteiro, Maria Paula Gomes Mourão, Marcus Vinícius Guimarães Lacerda

**Affiliations:** 1 Universidade do Estado do Amazonas, Manaus, Amazonas, Brazil; 2 Fundação de Medicina Tropical Dr. Heitor Vieira Dourado, Manaus, Amazonas, Brazil; 3 University of Massachusetts, Boston, Massachussetts, United States of America; 4 Instituto Leônidas & Maria Deane, Fiocruz, Manaus, Amazonas, Brazil; Kliniken der Stadt Köln gGmbH, GERMANY

## Abstract

Dengue fever is the most important arthropod-borne viral infection worldwide. Secondary prevention to reduce mortality through improved clinical case management has substantially lowered the mortality rate for severe dengue during the past two decades. Gallbladder wall thickening (GBWT) is a nonspecific finding often associated with more severe cases of dengue infection. This study had the aim to describe the ultrasonographic findings in hospitalized patients with dengue infection from Manaus (in the Western Brazilian Amazon) and to correlate the GBWT with dengue severity, symptoms and laboratorial analysis. Patients from 13–84 years admitted to the emergency department at the Fundação de Medicina Tropical Dr. Heitor Vieira Dourado (FMT-HVD) were enrolled in this study. Patients’ selection occurred during the most recent and huge dengue outbreak within the first semester of 2011. All enrolled subjects were systematically tested in order to rule out other possible etiologies for gallbladder inflammation. Abdominal ultrasound was performed by a single physician through bedside portable equipment and all other clinical and laboratorial information were retrieved from patients’ electronic files. 54 subjects were considered for analysis, with confirmed dengue infection by NS1 and/or RT-PCR positivity. From all enrolled patients, 50 (42.4%) presented GBWT. GBWT was significantly and independently related to: age under 31 years, pregnancy, presence of bleeding, presence of any cavitary effusion, DHF classification and severe dengue classifications. During dengue outbreaks, the GBWT identification through a non-invasive and bedside procedure is a confident marker for prompt recognition of potential severe cases.

## Introduction

Dengue virus (serotypes DENV-1 to DENV-4) is responsible for a self-limiting febrile illness and causes the highest disease burden of any arthropod-borne viral infection worldwide [1]. Recent studies estimate that there are 3.5 billion people living in areas of risk, contributing to over 390 million infections. Of these infections, 96 million manifest in dengue fever, 2 million in the severe disease, and 21,000 in death [2] [3]. Since the 1960s, there has been an increasing share of the disease burden in the Americas, especially Latin America, where a re-infestation of the dengue vector *Aedes aegypti* has brought infections up to 14% of the global rate in this region [3].

This re-emergence of infection, following a period of elimination in the Americas, reflects the limited success in primary prevention of dengue by vector control despite worldwide efforts (3). Furthermore, there is currently no highly efficacious vaccine or antiviral treatment. However, great progress has been made over the past 20 years in reducing mortality due to severe dengue from 10–20% to 1% through improved clinical management in a number of countries [4]. Thus, to determine which patients should be closely monitored, there is a case for refining criteria for early identification of patients at risk for developing severe dengue.

While symptomatic dengue fever generally resolves without major complications, some patients may progress to a more severe state even as the febrile phase abates. In 2009, World Health Organization dengue classification included dengue and severe dengue, the latter being characterized by severe plasma leakage, severe bleeding and/or organ impairment (1).

A main feature of severe dengue disease under both classifications is increased capillary permeability, represented by the escape of fluid and albumin into the extravascular space, leading to hemoconcentration, hypoproteinemia and cavitary effusions. Gallbladder wall thickening (GBWT) is one manifestation of increased capillary permeability [5]. GBWT above 3mm is significantly associated with more severe cases of dengue, and a thickness greater than 5mm could identify dengue patients with a higher risk of developing hypovolemic shock [6]. However, GBWT is a nonspecific finding related to many viral, bacterial and parasitological diseases [5].

The aim of this study was to associate GBWT with dengue severity, ruling out other etiologies, in hospitalized patients in a tertiary care unit from the Western Brazilian Amazon, where all four viral serotypes are currently circulating at the same time.

## Methods

### Ethical considerations

The study was reviewed and approved by the Ethics Review Board (ERB) of the *Fundação de Medicina Tropical Dr*. *Heitor Vieira Dourado* (protocol number 1718/2011). ERB waived the requirement for informed consent, as long as data were anonymized in the final databank.

### Study site and patient selection

This is a case series with hospitalized, acutely febrile patients in the Western Brazilian Amazon, consecutively enrolled.

All patients, regardless of sex, between 13–84 years of age admitted to the emergency department at the *Fundação de Medicina Tropical Dr*. *Heitor Vieira Dourado* (FMT-HVD) from March to April 2011 were enrolled in this study. FMT-HVD is a public health tertiary center for infectious diseases, located in Manaus, the capital of the western Brazilian Amazonas state.

Patient enrollment occurred during the largest dengue outbreak in Manaus, with more than 60,000 cases reported from January to May in 2011. This region present simultaneous circulation of all four DENV serotypes [7].

### Study design

This case series was conducted based on the review of clinical and laboratorial data from hospital files of patients admitted to emergency care. All these patients had systematic collection of clinical and laboratorial data according to a common and detailed protocol used during the outbreak. Patients were enrolled as cases after the review of: (1) hospital logbooks retrieved electronically from the computerized hospital records system (iDoctor), in which demographic data from each admitted patient, a presumptive diagnosis at admission and a final diagnosis at discharge are systematically registered; (2) hospital laboratory logbooks; and (3) abdominal ultrasound reports.

### Dengue classifications

Data of each admitted individual were systematically retrieved from the medical charts by the same member of the study team, and included the following variables: sex, age, duration of disease (in days) prior to the admission, outcome, presence of a given acute co-morbidity or chronic disease, presence of WHO-defined dengue warning signs and the final WHO-defined dengue classification.

Cases were categorized as dengue without warning signs (WS), dengue with WS, or severe dengue using WHO 2009 classification [8]. Dengue requires fever and two of the following: nausea, vomiting, rash, aches and pains, leukopenia, or any warning sign. Warning signs include abdominal pain or tenderness, persistent vomiting, clinical fluid accumulation, mucosal bleeding, lethargy or restlessness, hepatomegaly, or hematocrit rise (≥20%) with rapid drop in platelet count (≤100,000/mm^3^). Severe dengue includes severe plasma leakage, severe bleeding and/or severe organ impairment.

### Abdominal ultrasounds

A complete abdominal ultrasound was performed by a single physician through a bedside portable equipment–Sonosite 180 Plus (Bothell, Wisconsin, United States)–with a 3.5 MHz convex transducer, within the first 24 hours of admission.

On ultrasound, measurements were taken to determine the sizes of right and left liver lobes, thickness of anterior gallbladder wall, parietal surface thickness, and spleen size. Liver enlargement was defined as follows: the right lobe having a longitudinal diameter in the mid-hemiclavicular line to the right portal vein >15cm and the left lobe with a longitudinal diameter in the median line >10cm. Cavitary effusions (pleural and abdominal) were identified and hepatic and splenic textures were characterized. In addition, major and minor criteria for cholecystitis were evaluated for each patient according to guidelines defined by Huffman et al [9]. The blinded measurements of each variable were taken and the average between them was considered for analysis.

### Laboratory tests

Confirmed dengue infection was assumed for subjects with positive NS1 (*Platelia*^™^ *Dengue NS1 Ag Kit*, Bio-Rad Laboratories, France) or RT-PCR blood assays [10]. Presumptive dengue infection was considered for subjects with positive IgM (MAC-ELISA) in a single acute-phase sample [11].

In order to rule out other possible etiologies for GBWT, all enrolled subjects were systematically tested for malaria (thick blood smear), HIV (antigen detection through rapid test), *Toxoplasma gondii* (ELISA IgM and IgG), Epstein-Barr virus (ELISA IgM and IgG), hepatitis C (total anti-HCV), hepatitis B (HBsAg), typhoid fever (blood culture) and leptospirosis (ELISA IgM).

### Statistical analysis

Data were analyzed using SPSS version 16.0 for Windows (SPSS Inc. Chicago, IL, USA). Differences in proportion were calculated if p<0.05 in the Fisher exact test (corrected by Yates’ test if necessary). The crude *Odds Ratio* (OR) with its respective 95% Confidence Interval (95% CI) was determined. Logistic regression was used for the multivariate analyses and the adjusted OR with 95% CI were also calculated. A multivariate logistic regression was performed with severe dengue as the outcome, using an automated backward and forward stepwise estimation. All variables that were associated with GBWT at a significance level of p<0.10 in the univariate analysis were included in the initial model. Statistical significance was considered if p<0.05 in the Hosmer-Lemeshow goodness-of-fit test.

## Results

**Fig 1:**
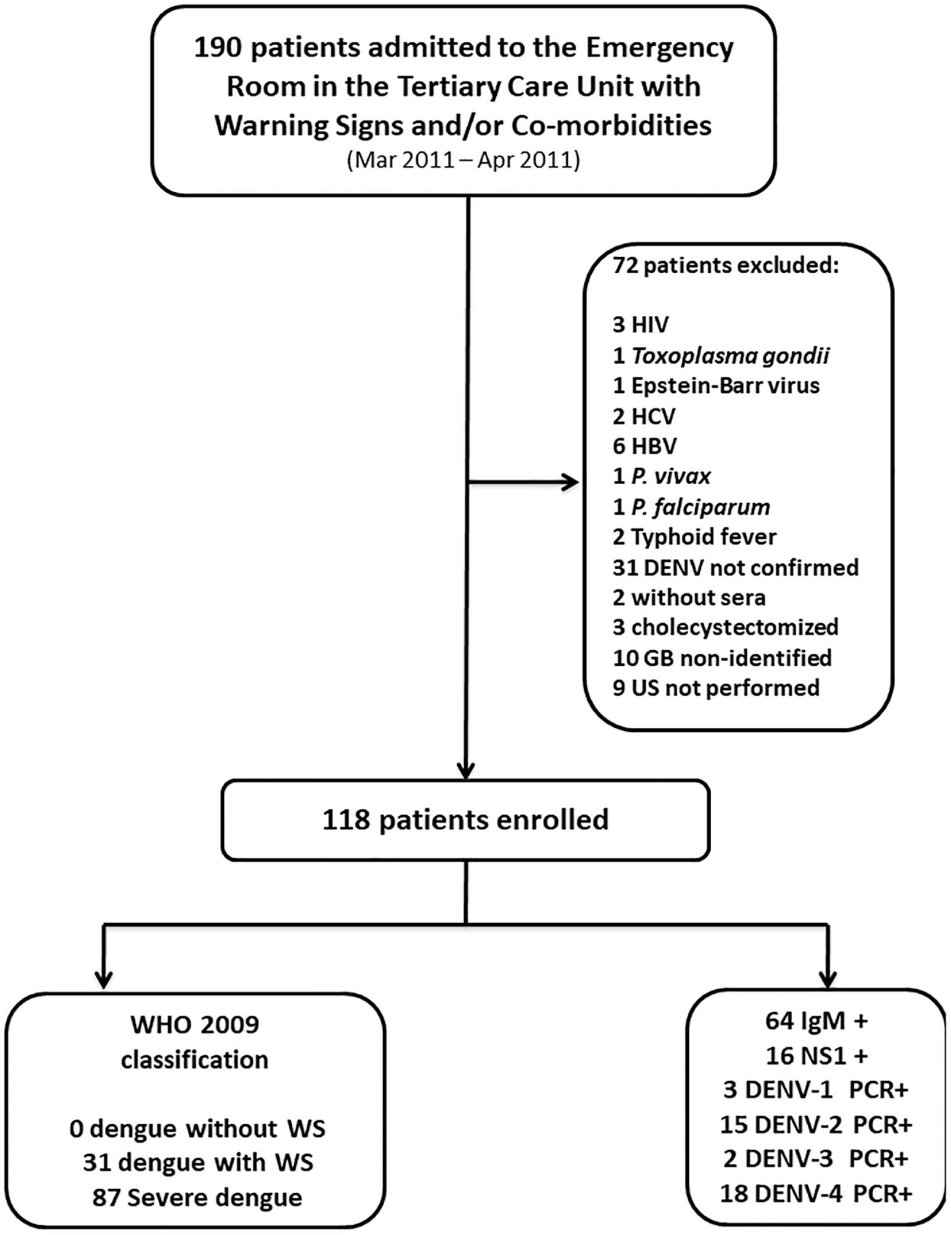
Breakdown of patient enrollment, exclusions and classifications.

From March to April 2011, 190 patients were enrolled in the study as described in Fig 1. Seventy-two (37.9%) patients were later excluded after systematic testing showed concomitant infections, due to lack of dengue infection confirmation, no identification of gallbladder during abdominal ultrasound, or inability to obtain ultrasound. The remaining 118 subjects were considered for analysis: 54 with confirmed dengue infection and 64 with presumptive dengue infection. PCR analysis was available for 38 of the 54 confirmed cases, and demonstrated the presence of all four serotypes during the 2011 dengue outbreak in the Western Amazonian region.

These 118 patients were classified using WHO 2009 guidelines and clinical, laboratorial, and ultrasonographic findings. 31 patients presented dengue with warning signs and 87 presented severe dengue. Females comprised 95 (80.5%) of the cases and the median age was 31 years, with a range from 13–84. A previous co-morbidity or chronic disease was present in 27 (22.9%) patients, with arterial hypertension being the most frequent (74.1%).

The overall ultrasonographic findings are described in Table 1. Of the 2009 WHO-classified dengue warning signs measurable by US, GBWT was the most frequent finding (42.4%) in this study, followed by cavitary effusions (33%) and liver right lobe enlargement (10%). There was no significant difference in ultrasonographic findings between patients with confirmed and presumptive dengue (data not shown).

**Table 1:** Epidemiological and clinical parameters between confirmed and presumptive dengue virus-infected patients with warning signs, without and with gallbladder wall thickening (Manaus, Brazil, 2011).

Variable	Patients without GBWT (N = 68) n (%)	Patients with GBWT (N = 50) n (%)	Unadjusted OR (CI95%)	p-value*	Adjusted OR*** (CI95%)	p-value
Male gender	28 (41.2)	25 (50.0)	1.21 (0.82–1.80)	0.34	-	-
Age ≤ 31 years	27 (39.7)	33 (66.0)	1.66 (1.16–2.36)	**<0.01**	-	-
Pregnancy	0 (0.0)	4 (16.0)	-	0.02**	-	-
≥ 5 days of fever duration	46 (67.6)	41 (82.0)	1.21 (0.98–1.49)	0.08	-	-
Jaundice	2 (2.9)	2 (4.0)	1.36 (0.19–9.32)	0.75	-	-
Persistent vomiting	13 (19.1)	6 (12.0)	0.62 (0.25–1.53)	0.30	-	-
Severe abdominal pain	44 (64.7)	37 (74.0)	1.14 (0.89–1.45)	0.28	-	-
Bleeding	46 (67.6)	44 (88.0)	1.30 (1.07–1.57)	**0.01**	-	-
Liver enlargement (right lobe) by ultrasound	8 (11.8)	10 (20.0)	1.70 (0.72–3.99)	0.22	-	-
Liver enlargement (left lobe) by ultrasound	2 (2.9)	2 (4.0)	1.36 (0.19–9.32)	0.75	-	-
Spleen enlargement by ultrasound	6 (8.8)	5 (10.0)	1.13 (0.36–3.50)	0.82	-	-
Any cavitary effusion by ultrasound	13 (19.1)	26 (52.0)	2.72 (1.55–4.74)	**<0.01**	-	-
Ascitis	5 (7.4)	15 (30.0)	4.08 (1.58–10.48)	**<0.01**	-	-
Pleural effusion	13 (19.1)	21 (42.0)	2.19 (1.22–3.95)	**<0.01**	-	-
Severe dengue (WHO, 2009)	45 (66.2)	42 (84.0)	1.26 (1.03–1.56)	**0.03**	2.84 (1.09–7.38)	**0.03**

GBWT: Gallbladder Wall Thickening; OR: Odds Ratio; CI95%: Confidence Interval 95%; SD: Standard Deviation;

* Chi-squared test;

** Fisher test;

*** Modelling for logistic regression was performed individually using both dengue classifications, adjusted for age and duration of fever.

**Fig 2:**
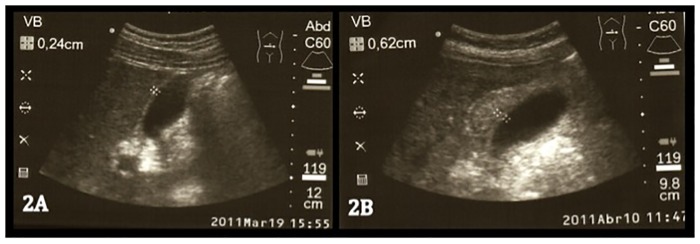
Abdominal ultrasound in a control patient (2A) and in a patient with severe dengue (2B).

From all enrolled patients with confirmed or presumptive dengue, 50 (42.4%) presented GBWT at admission and/or within 24 hours of admission. This number constituted 84% of severe dengue subjects. Fig 2 shows a sample of an abdominal US picture from a control patient (2A) compared with a patient with severe dengue (2B).

Epidemiological and clinical parameters of both confirmed and presumptive dengue patients are described in Table 2. Under WHO 2009 classification, severe dengue developed more frequently among patients with GBWT (p = 0.03). GBWT was also significantly and independently related to: age ≤ 31 years, pregnancy, presence of bleeding, and the presence of any cavitary effusion. On the other hand, GBWT did not show any relation to biochemical blood tests (liver enzymes, albumin, DHL, FAL, GGT,) or hematological parameters (hemoconcentration and platelet count), in this study.

**Table 2:** Overall ultrasonographic findings in dengue virus-infected patients with warning signs (Manaus, Brazil, 2011).

Variable	Dengue confirmed cases (n = 54) n (%)	Dengue presumptive cases (n = 64) n (%)	Total (N = 118) n (%)
Liver right lobe enlargement	8 (14.8)	10 (15.6)	18 (10.0)
Liver left lobe enlargement	4 (7.4)	0 (0.0)	4 (3.4)
Liver steatosis	5 (9.2)	6 (9.4)	11 (9.3)
Spleen enlargement	6 (11.1)	0 (0.0)	6 (5.1)
Gallbladder wall thickening			
>3mm	21 (38.9)	29 (45.3)	50 (42.4)
>5mm	17 (14.4)	25 (39.1)	42 (35.6)
GBWT pattern			
Striated	11 (20.4)	13 (20.3)	24 (20.3)
Double layer	5 (9.2)	6 (9.4)	11 (9.3)
Asymmetric	1 (1.8)	2 (3.1)	3 (2.5)
Uniform	1 (1.8)	1 (1.6)	2 (1.7)
Pericholecystic fluid	5 (9.2)	8 (12.5)	13 (11.0)
Cavitary effusions	15 (27.8)	24 (37.5)	39 (33.0)
Pleural effusion	14 (25.9)	20 (31.2)	34 (28.8)
Ascites	8 (14.8)	12 (18.7)	20 (16.9)

GBWT: Gallbladder Wall Thickening; mm: milimeters.

**Fig 3:**
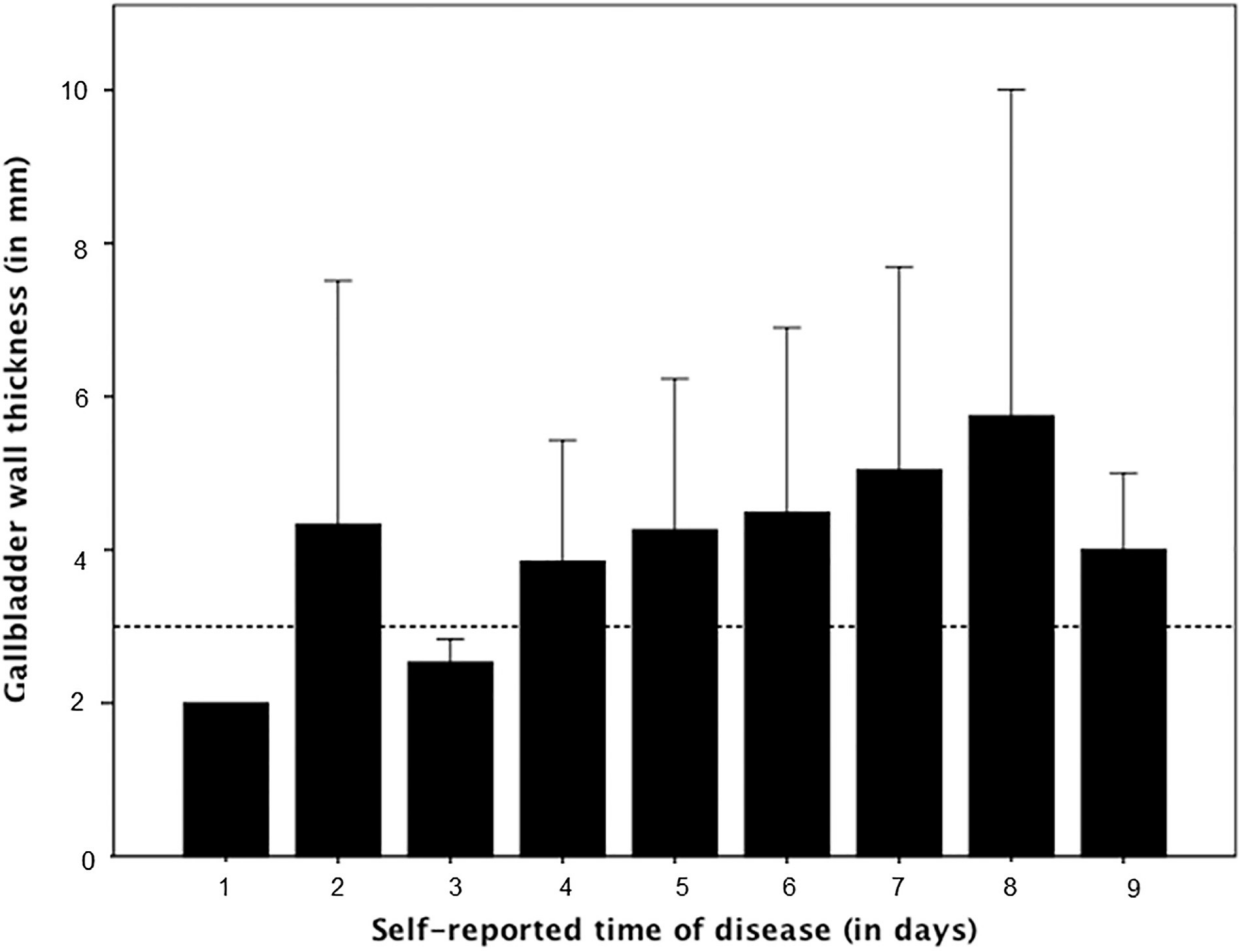
Measurements of GBWT in each individual patient according to the day of referred disease when the ultrasound was performed.

In general, measurements of GBWT showed a progressive increase between days 3 to 8 of illness, the critical phase of dengue fever, as shown in Fig 3. All enrolled patients developed a complete recovery during hospitalization and no fatalities or need for intensive care management were observed in this case series.

## Discussion

This study shows the correlation between GBWT and dengue severity. Several groups have demonstrated this correlation in the past [12], and a recent study in Indonesia has conducted a prospective cohort study showing increased GBWT during severe dengue, and reduced GBWT after resolution of the illness [13]. Still, the current frequency of GBWT in dengue patients is uncertain. Previous studies have reported rates ranging from 29.8% [14] to 100.0% [15]. However, these numbers were primarily derived from small case series of presumptive dengue infected patients (diagnosed only by serology). Our study findings are more robust because the results come from a large patient sample size (118 patients with confirmed (NS1 and/or RT-PCR) or presumptive [single sample positive IgM] dengue infection), with data gathered from a region in the Amazon where the four dengue serotypes are concomitantly present, while specifically excluding patients with other etiologies that are known to contribute to GBWT [5]. In patients with dengue, we found that the overall frequency of GBWT was 42.5%.

When severe dengue is identified, careful monitoring in a hospital setting can reduce mortality. In this study, the GBWT showed a progressive increase between days 3 to 8 of illness, corresponding to the critical phase of dengue fever [16]. Because there are no current widely accepted biomarkers, the detection of GBWT by ultrasound in the beginning of the critical phase could be a means of detecting severe dengue.

In addition, it is important to correlate GBWT with possible dengue infection as GBWT can sometimes be misdiagnosed as acute acalculous cholecystitis. This may lead to unnecessary cholecystectomy and the associated complications of this surgery (e.g. severe spontaneous bleeding, blood components transfusion, prolonged hospitalization, or death [17,18].

In the present study, the GBWT did not show any relation to biochemical blood tests (liver enzymes, albumin, DHL, FAL, GGT) or hematological parameters (hemoconcentration and platelet count), these findings differ from the results of previous studies [17,19,20]. It is important to notice that, in this case series, thrombocytopenia (platelet count ≤ 100,000/mm^3^) was assumed as a hospitalization criteria when accompanied by any of the already described warning signs, leading to a very high frequency (98%) among our hospitalized patients (data not shown). Hemoconcentration, once thought to be significantly linked to increased dengue severity, appears unlinked to dengue severity as measured by plasma leakage [13] and GBWT. Hemoconcentration (≥20% variation between admission and discharge) was an infrequent event (10.0% in GBWT and 13.2% non-GBWT) among adult hospitalized patients. GBWT was also found to be independent of liver enlargement.

Finally, no fatalities or need for intensive care management were observed in this case series from Manaus (Brazil). This should be attributed to massive and repeated training of both public and private health system personnel, from all different levels of attention, in order to provide high quality of care and prompt identification of potential severe cases [14].

Our study has limitations. First, because of the size and cost limitations of our study, we were unable to conduct a longitudinal study and perform follow-up ultrasounds on our patients after they were discharged. This would be helpful to confirm, as others have done [13] that the GBWT lessens after the dengue subsides, demonstrating that the GBWT was a transient consequence of the dengue infection. Second, patients were referred to the emergency department of the tertiary care facility. This meant that we did not enroll our patients until many were in the critical phase of dengue symptoms. Therefore, we cannot conclude at what stage in the dengue disease process GBWT begins. It is needed to perform a study looking at patients earlier in their dengue infection to see if GBWT can be used as an early marker for potential development of severe dengue. Third, although patients were instructed to fast before their ultrasound, we cannot rule out that some patients may have eaten and this may contribute to the appearance of GBWT in some patients. However, it is likely that patients who ate did so randomly between both arms of the study (severe and non-severe dengue patients), so this did not likely contribute largely to our results. Fourth, although we enrolled all patients who displayed comorbidities or warning signs of dengue, regardless of sex, 80.5% of the patients enrolled in our study were women. We believe that this is because women are more likely than men to develop severe dengue [21] in addition to being more likely to acknowledge symptoms and seek medical care [22].

In conclusion, the findings of our study highlight the need for careful monitoring of dengue patients in the Western Amazonian region in Brazil, where all four dengue serotypes are confirmed to be circulating. By systematically ruling out other etiologies for GBWT in a large cohort of patients during a major dengue outbreak, we have also shed light on the relationship between GBWT and dengue severity. Building on previous works that have demonstrated the effectiveness and cost-effectiveness of ultrasonography in evaluating cavitary effusions, our study may contribute to more deliberate management of dengue patients to identify severe cases while avoiding misdiagnoses.

## Supporting information

S1 Table Study database. (XLSX)
